# Matrine Inhibits High-Glucose-Diet-Induced Fat Accumulation and Aβ-Mediated Lipid Metabolic Disorder via AAK-2/NHR-49 Pathway in *Caenorhabditis elegans*

**DOI:** 10.3390/ijms26073048

**Published:** 2025-03-26

**Authors:** Aimin Qiao, Meiqing Pan, Yue Zeng, Ying Gong, Yunfeng Zhang, Xiucai Lan, Lei Tang, Weizhang Jia

**Affiliations:** School of Life Sciences and Biopharmaceutics, Guangdong Pharmaceutical University, Guangzhou 510006, China; qiaoam2014@163.com (A.Q.); panmq2022@163.com (M.P.); zy2674132099@163.com (Y.Z.); gy13234492469@163.com (Y.G.); willsonzyf713@gmail.com (Y.Z.); lxc2024124@163.com (X.L.); ashoretl@163.com (L.T.)

**Keywords:** matrine, high-glucose diet, fat accumulation, Aβ-mediated lipid metabolic disorder, AAK-2/NHR-49 pathway

## Abstract

Matrine, a quinoline alkaloid, possesses lipid-regulating effects, but the underlying mechanisms are rarely characterized in vivo. With a fat-accumulating *Caenorhabditis elegans* model, we show that matrine reduces the fat content and the DHS-3::GFP-labeled lipid droplets in high-glucose-diet N2 and transgenic LIU1 nematodes, respectively. Based on RNA-seq, this study demonstrates that a loss of AAK-2 function suppresses the fat-lowering effects of matrine, and the hyperactivated AAK-2 strain has a relatively lower fat content than N2. The involvement of NHR-49 in matrine’s fat-lowering effects further suggests that matrine impacts fat breakdown and storage via the AAK-2/NHR-49-governed pathway. Using the transgenic SJ4143 (*ges-1::GFP(mit)*) and VS10 (*vha-6p::mRFP-PTS1*), we show that matrine activates the AAK-2/NHR-49 pathway, coupling the alteration of mitochondrial and peroxisomal functions. Studies of *aak-2* and *nhr-49* mutants reveal that AAK-2 and NHR-49 modulate lipid metabolic homeostasis; meanwhile, matrine increases physical fitness and lifespan through activating the AAK-2/NHR-49 pathway in high-glucose-diet nematodes. Surprisingly, we found that β-amyloid (Aβ) induces lipid metabolic disorder in an Alzheimer’s disease (AD) *C. elegans* model, but matrine not only reduces Aβ aggregation but also alleviates Aβ-mediated lipid metabolic disorder. Our data suggest that matrine has promise as a fat-lowering agent, and also offer new insights into its therapeutic potential for AD.

## 1. Introduction

Obesity is closely related to unbalanced energy intake and expenditure, which cause the body to convert excess energy into fatty acids and store them in adipose tissue [1]. With the alteration of dietary patterns in modern society, the prevalence of obesity, as measured by body mass index (≥30 kg/m^2^) [2], has increased significantly globally and become a serious problem threatening human health. Obesity increases the incidences of hyperlipidemia, non-alcoholic fatty liver, type II diabetes, and heart diseases, as well as shortened lifespans and multiple forms of cancer [3]. In addition to the known toxic effects of excess fatty acids, adipose tissue dysfunctions, including lipid metabolic disorders, hormonal abnormalities, and a pro-inflammatory condition, have become vital factors in the occurrence of obesity-associated diseases [2]. Although various strategies are used in attempts to control weight, including diets, physical activities, and behavioral therapies, using fat-lowering agents to manage obesity is beneficial to improving existing lipid metabolic disorders and inflammatory reactions. Research shows that natural active substances, such as berberine and coptisine, inhibit fat accumulation and alleviate lipid metabolic disorder and inflammatory response [4,5]. Thus, finding fat-lowering compounds from natural products is urgently required to treat obesity and its comorbidities.

Convincing evidence shows that midlife obesity is epidemiologically linked to increased risks of neurodegeneration and Alzheimer’s disease (AD) [6]. Identification of lipid-related susceptibility loci such as *apolipoprotein E* (*ApoE*) indicated that the dysfunction of lipid metabolism favored the pathogenesis of AD, and the perturbation of fatty acid profiles also affected AD progression by promoting the aggregation of amyloid-beta (Aβ) and Tau [6]. Thus, natural active substances with lipid-lowering and anti-inflammatory effects also displayed therapeutic benefits against age-related neurodegenerative diseases [7,8]. The mechanisms underlying the lipid-regulating effects partly involve the activation of AMP-activated protein kinase (AMPK) and nuclear receptors such as peroxisome proliferator-activated receptor α (PPARα), which then modulate glycolipid metabolism, inflammation, and energy homeostasis [9,10]. For example, berberine inhibits the production and accumulation of Aβ in AD mice, and rescues cognitive decline through the activation of the AMPK-related pathway in aging rats [8,11]. AMPK is therefore considered an important molecular target, as it is believed that novel AMPK activators might be used for the treatment of lipid metabolic disorders and neurodegenerative diseases [12]. In summary, supplementation with fat-lowering bioactive substances is beneficial to obese individuals and reduces the risk of obesity-related complications.

Matrine, a naturally occurring quinoline alkaloid extracted from dried roots and fruits of Leguminosae *Sophora flavescens* Ait., possesses multiple biological effects, including anti-inflammatory, antioxidative, antifibrotic, and immune function-improving effects [13,14]. Matrine also exhibited the capacity for lipid-regulating and neuroprotective effects. For example, matrine decreases lipid levels by inducing the production of low-density lipoprotein (LDL) receptors and enhancing LDL uptake in HepG2 and A549 cells, and mitigates obesity in high-fat-diet mice [15,16]. In addition, Cui et al. found that matrine inhibited Aβ_42_-induced cytotoxicity in vitro, reduced pro-inflammatory cytokines and Aβ aggregation, and alleviated the memory decline of transgenic AD mice [17]. In the present study, the nematode *Caenorhabditis elegans*, a popular model for investigating lipid metabolism and human diseases, was selected to explore the mechanisms by which matrine inhibited high-glucose diet-induced fat accumulation and Aβ-mediated lipid metabolic disorder. Our results show that matrine reduces fat accumulation and improves lipid metabolic disorder via the AAK-2/NHR-49-governed pathway, which highlights its promise as a fat-lowering agent and also offers new insights into its therapeutic potential for AD.

## 2. Results

### 2.1. Matrine Reduces Fat Accumulation in High-Glucose-Diet C. elegans

**Figure 1:**
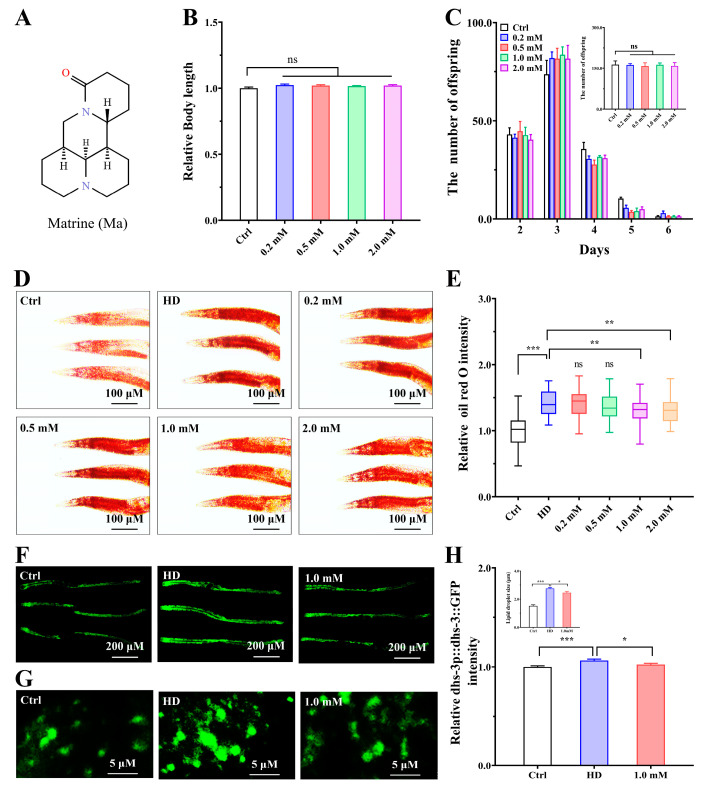
Matrine reduces fat accumulation in high-glucose-diet nematodes. (**A**) Molecular formula of matrine. (**B**) Effect of matrine on body length. Wild-type L4-stage *C. elegans* were incubated with matrine (0, 0.2, 0.5, 1.0, and 2.0) for 48 h and then their body lengths were measured. (**C**) Effect of matrine on brood size (*N* = 5 animals per group). Wild-type nematodes were treated as described in (**B**) and transferred individually to new NGM plates daily. The parents were removed after oviposition, and the offspring were counted. (**D**) Oil red O staining of N2 nematodes. L1-stage nematodes were fed on NGM plates with or without glucose (10 mM) and supplemented with different concentrations of matrine (0, 0.2, 0.5, 1.0, and 2.0 mM) for 72 h. (**E**) Quantification of fat content. (**F**,**G**) Fluorescent images of LIU1 (*dhs-3p::dhs-3::GFP*) nematodes. Synchronized LIU1 nematodes were incubated on normal or high-glucose NGM plates supplemented with matrine (0 and 1.0 mM) for 48 h. (**H**) Quantification of DHS-3::GFP fluorescent intensity and lipid droplet size in LIU1 nematodes. The results were presented as mean ± SEM (*n* = 50, two independent trials), ns: not significant, *: *p* < 0.05, **: *p* < 0.01, ***: *p* < 0.001.

Matrine, a tetracyclic quinoline alkaloid found in *Sophora flavescens*, used in traditional Chinese medicine (Figure 1A), has been used for the treatment of hyperlipidemia, type II diabetes, and hepatic steatosis [15,16]. Here, we investigated the fat-lowering effect and the mechanism of matrine in a high-glucose-diet-induced fat-accumulating *C. elegans* model. First, we investigated the impact of matrine on the growth and brood size of N2 *C. elegans*. When matrine (0, 0.2, 0.5, 1.0, and 2.0 mM) was used to incubate with *C. elegans*, there were no statistical differences in body length and offspring (Figure 1B,C). In order to observe matrine’s fat-lowering effect, we used Oil red O (a strong fat solvent) staining to detect the fat content in the intestines of N2 nematodes [18]. The staining results showed that supplementation with 10 mM glucose significantly increased the fat content of nematodes, confirming the successful establishment of the high-glucose-diet-induced fat-accumulating nematode model (Figure 1D) [19]. At the same time, we found that the fat content in N2 nematodes was reduced in a dose-dependent relationship with an increase in matrine concentration from 0.2 to 2.0 mM (Figure 1E). It was worth noting that the high-glucose-diet nematodes administered with 1.0 mM matrine had a lower fat storage compared to the other groups. To further validate the fat-lowering effects of matrine, we employed the LIU1 strain (*dhs-3p::dhs-3::GFP*) containing the DHS-3 protein fused to GFP, which was selected as a marker of intestinal lipid droplets in nematodes [20]. The results showed that the high-glucose diet increased the fluorescent intensity and the lipid droplet size in LIU1 nematodes. In contrast, supplementation with matrine (1.0 mM) reduced the increased fluorescent intensity and lipid droplet size (Figure 1F–H). Taken together, our study indicates that matrine has the capacity to reduce fat accumulation and thus possesses fat-lowering activity.

### 2.2. Transcriptome Analysis of Matrine-Regulated DEGs in High-Glucose Diet C. elegans

**Figure 2:**
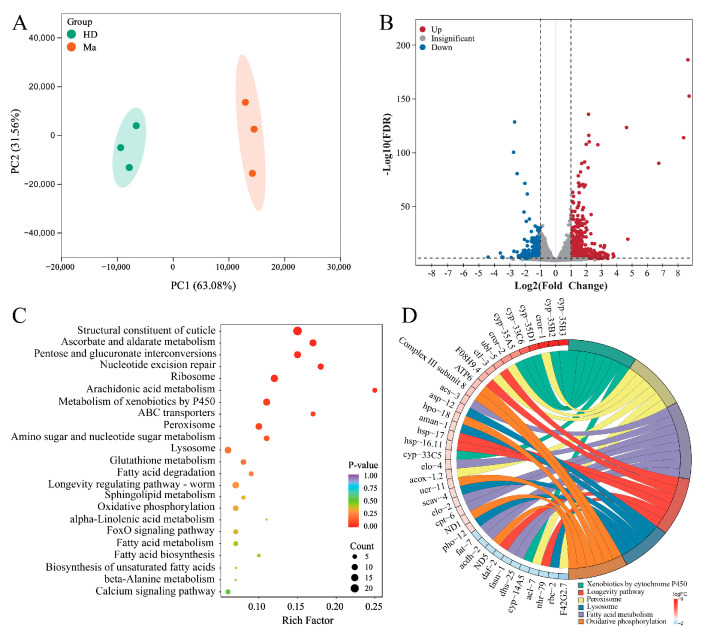
Transcriptome analysis of matrine-regulated DEGs in high-glucose-diet *C. elegans*. (**A**) Principal component analysis (PCA) of sequencing samples. Synchronized L1-stage N2 nematodes were fed with matrine (0 and 1.0 mM) for 48 h and then collected for RNA sequencing and bioinformatic analysis. (**B**) DEG volcano plots for high-glucose-diet nematodes treated with or without matrine. The vertical dashed lines are the threshold for DEGs. The horizontal dashed line represents the −log10(FDR) threshold. The DEGs were identified at thresholds of FC ≥ 2 and FDR < 0.05, with 706 genes being upregulated and 340 genes being downregulated. (**C**) KEGG enrichment of DEGs. (**D**) The chord diagram of DEGs. The designated DEGs regulated by matrine are clustered into 6 classifications in accordance with molecular function, including fatty acid metabolism, xenobiotic by cytochrome P450, longevity pathway, peroxisome, lysosome, and oxidative phosphorylation, and many of the DEGs contain NHR-49 binding sites (Table S1).

To understand matrine-mediated regulatory networks, high-glucose-diet N2 nematodes administered with or without matrine were used to carry out transcriptome sequencing (RNA-seq). We employed principal component analysis (PCA) to evaluate the variation and correlation in RNA-seq *C. elegans* samples [21]. The results show that the samples in the control and matrine-treated groups have good repeatability within the group, but there is a good discrimination between the two groups (Figure 2A). With a filter of false discovery rate (FDR) < 0.05 and fold change (FC) ≥ 2.0, a total of 1046 differentially expressed genes (DEGs) were discovered between the matrine-treated and control groups, including 706 upregulated and 340 downregulated genes (Figure 2B). The Kyoto Encyclopedia of Genes and Genomes (KEGG) analysis displays that DEGs are mainly enriched in structural constituent of cuticle, glycolipid metabolism, xenobiotic metabolism, peroxisome, lysosome, and oxidative phosphorylation (Figure 2C). Subsequently, the designated DEGs of the six terms were represented in a chord diagram (Figure 2D), in which the upregulated DEGs were mainly related to xenobiotics, peroxisome, lysosome, and lipolysis pathways, while the downregulated DEGs were mainly related to lipogenesis. It is worth noting that the promoter regions of most of these DEGs contain NHR-49 binding sites (Figure S1), indicating that they may be the target genes of NHR-49 transcription factor (Table S1). In summary, RNA-seq analysis suggests that the fat-lowering effects of matrine may be achieved by regulating related target genes through the NHR-49-related pathway.

### 2.3. Matrine-Mediated Fat Lowering Through AAK-2 Pathway but Not DAF-16

**Figure 3:**
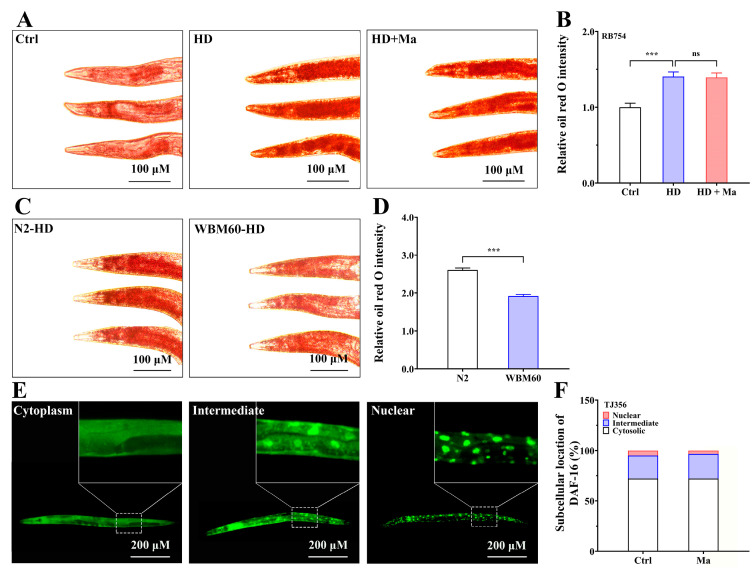
Matrine-mediated fat lowering through AAK-2 pathway but not DAF-16. (**A**) Oil red O staining of RB754 (*aak-2* mutant) nematodes. L1-stage RB754 nematodes were fed for 24 h, and then placed on new NGM plates with matrine (0 and 1.0 mM) for 48 h. (**B**) Quantification of fat content. (**C**) Oil red O staining of N2 and WBM60 (hyperactivated AAK-2) nematodes. L1-stage N2 and WBM60 nematodes were fed for 24 h, and then transferred to normal or high-glucose-diet plates for 48 h. (**D**) Quantification of fat content. (**E**) Images of DAF-16::GFP localization with “cytoplasm”, “intermediate”, and “nuclear” in TJ356 nematodes. L1-stage TJ356 nematodes were incubated with matrine (0 and 1.0 mM) for 48 h. The image of the local magnification is displayed in the upper right corner. (**F**) Proportion of nematodes with DAF-16::GFP localizing in “cytoplasm”, “intermediate”, and “nucleus”, respectively. Results are presented as mean ± SEM (*n* = 50, two independent trials). ns: not significant, ***: *p* < 0.001.

In nematodes, AAK-2 is the homolog of the human AMPK α-subunit, which is conserved among eukaryotes and is involved in maintaining the homeostasis of lipid metabolism [22]. Since AMPK, a hub of metabolic regulation, has become an important therapeutic target for obesity, we investigated whether matrine exerted its fat-lowering effects in high-glucose-diet nematodes through the AMPK pathway. Using the loss-of-function *aak-2* mutant (RB754, *aak-2(ok524)*), our study found that a high-glucose diet significantly enhanced fat storage. However, matrine supplementation did not reduce the fat content in high-glucose-diet RB754 mutants (Figure 3A,B), suggesting that matrine exerts fat-lowering effects through the AAK-2 pathway. To further determine the effect of AAK-2 on fat storage, we conducted Oil red O staining on WBM60 (hyperactivated AAK-2) nematodes. As presented in Figure 3C,D, the fat storage of WBM60 nematodes was lower than that of N2 nematodes under the high-glucose diet condition, suggesting that hyperactivated AAK-2 promotes lipolysis and reduces fat storage in nematodes. In conclusion, these results indicate that the AAK-2/AMPK pathway participates in the fat-lowering effect of matrine in *C. elegans*.

Moreover, DAF-16 is the homolog of human FoxO regulator in nematodes, and it is the main downstream component of the insulin/IGF-1 signaling (IIS) pathway that integrates various signal pathways, including metabolism, aging, and immunity [23]. To evaluate whether matrine activates DAF-16, we investigated the nuclear localization of DAF-16 in TJ356 nematodes (DAF-16A/B fused to GFP), and the fat content in CF1038 nematodes (*daf-16* loss-of-function mutant). As presented in Figure 3E, the subcellular localization of DAF-16 could be divided into three categories: cytoplasm, intermediate, and nuclear. However, matrine treatment did not increase the proportion of nuclear DAF-16::GFP (Figure 3F). Moreover, matrine was able to reduce lipid storage in *daf-16* mutant nematodes (Figure S2), suggesting that matrine is independent of DAF-16 for its fat-lowering effect. Taken together, our study demonstrates that the fat-lowering effects of matrine do not depend on DAF-16, but involve the AAK-2/AMPK pathway.

### 2.4. Involvement of NHR-49 in Matrine-Mediated Fat-Lowering Effect

**Figure 4:**
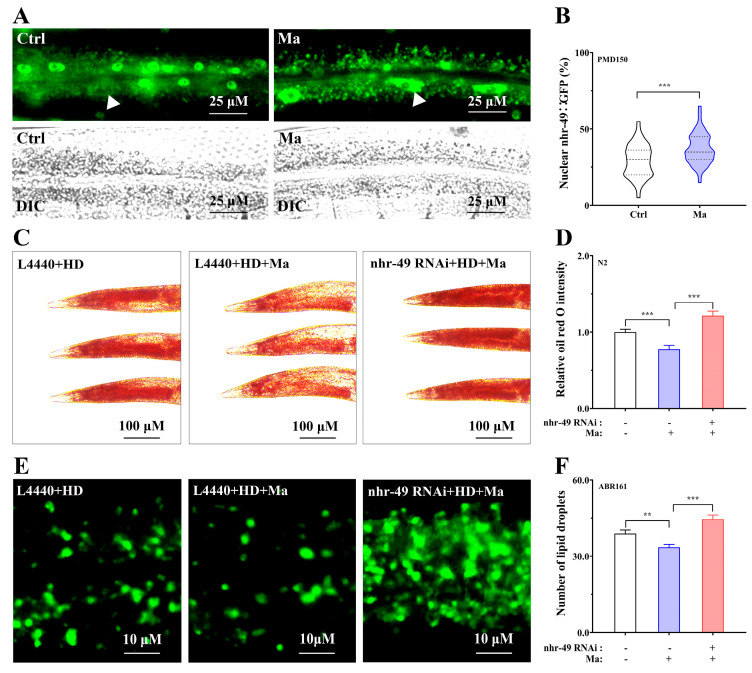
Involvement of NHR-49 in matrine-mediated fat-lowering effect. (**A**) NHR-49::GFP localization in PMD150 (*nhr-49p::nhr-49::GFP*) nematodes. L1-stage PMD150 nematodes were incubated for 24 h, and then treated with matrine (0 and 1.0 mM) for 48 h. Subsequently, the subcellular localization of NHR-49::GFP was observed. Top panel: Representative fluorescent images. Bottom panel: Differential interference contrast (DIC) images. White arrows indicate the position of intestinal nucleus. (**B**) Proportions of intestinal cells exhibiting nuclear-localized NHR-49::GFP. The thick dashed line is the median. The upper thin dashed line is the third quartile (Q3), and the lower thin dashed line is the first quartile (Q1). (**C**) Oil red O staining of high-glucose-diet N2 worms treated with or without *nhr-49* RNAi. Nematodes were fed for 24 h with HT115 carrying *L4440* or *nhr-49* recombinant, and then placed on high-glucose plates coated with HT115 (containing *L4440* or *nhr-49* recombinant) and matrine (0 and 1.0 mM) for 48 h. (**D**) Quantification of fat content. (**E**) Fluorescent images of high-glucose-diet ABR161 worms treated with or without *nhr-49* RNAi. ABR161 nematodes were treated with the experimental program in (**C**) for counting lipid droplets. (**F**) Quantification of lipid droplets in ABR161 nematodes. The results were shown as mean (*n* = 50, two independent trials). **: *p* < 0.01, ***: *p* < 0.001.

Previous research shows that AAK-2 may regulate the activation of NHR-49, the sequence and function related to mammalian peroxisome proliferator-activated receptor *α* (PPARα), to impact the catabolism of fatty acids [24]. In order to investigate the fat-lowering mechanism of matrine, we evaluated the effect of matrine on the activation of NHR-49 using the transgenic PMD150 nematodes expressing *nhr-49p::nhr-49*::GFP reporter. As presented in Figure 4A,B, the NHR-49::GFP signal was relocalized to the intestinal epithelial nucleus after matrine treatment, resulting in an increase in the proportion of worms exhibiting high levels of nuclear NHR-49::GFP. Thus, matrine could impact the transcriptional activity of NHR-49, thereby altering the expression profile of fat metabolic genes. To further analyze the impact of NHR-49 on the activity of matrine, we examined the fat-lowering effect of matrine after *nhr-49* RNAi in N2 nematodes and the intestinal lipid droplets in transgenic ABR161 nematodes (expressing *dsh-3p::dhs-3::GFP* reporter), respectively. Using Oil red O staining in N2 nematodes, our results showed that *nhr-49* RNAi not only eliminated the fat-lowering effect of matrine, but also led to more significant fat accumulation, suggesting that NHR-49 is involved in the matrine-induced fat-lowering effect (Figure 4C,D). Consistent with this, *nhr-49* RNAi significantly increased the number of DHS-3::GFP-labeled intestinal lipid drops in high-glucose-diet ABR161 nematodes (Figure 4E,F). Therefore, we infer that matrine reduces the fat accumulation of high-glucose-diet *C. elegans* in an NHR-49/PPARα-dependent manner.

### 2.5. Matrine Regulates Lipid Metabolism-Related Gene Expressions

**Figure 5:**
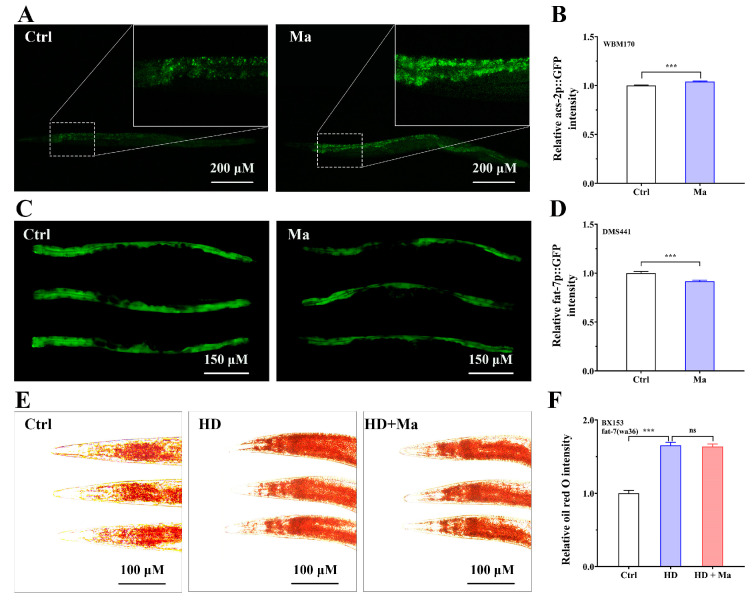
Matrine regulates lipid metabolism-associated gene expressions. (**A**) Fluorescent images of WBM170 (*acs-2p::GFP*) nematodes. L1-stage WBM170 were fed for 24 h and then placed on new NGM plates with matrine (0 and 1.0 mM) for 48 h. The local magnification is displayed in the upper right corner. (**B**) Quantitative analysis of *acs-2p::GFP* fluorescent intensity. (**C**) Representative images of DMS441 (*fat-7p::GFP*) nematodes. The DMS441 nematodes were treated with the experimental program in (**A**) for detecting the fluorescent intensity. (**D**) Quantitative analysis of *fat-7p::GFP* fluorescent intensity. (**E**) Oil red O staining of BX153 (*fat-7* mutant (*wa36*)) nematodes. The BX153 nematodes were treated as shown in (**A**) and then the fat content was measured. (**F**) Quantification of fat content. These results were shown as the mean (*n* = 50 two independent trials). ns: not significant, ***: *p* < 0.001.

Study shows that metabolic networks accurately control fat storage by synthesis and breakdown pathways, and that the degree of obesity in an animal depends on the rate of fatty acid decomposition as well as the synthetic rate of fatty acids [25]. Therefore, the genes such as *acs-2* and *fat-7* encoding the molecular components of these metabolic processes may underlie matrine’s fat-lowering effects. Using the transgenic WBM170 nematodes (expressing *acs-2p*::GFP reporter), we intended to detect the expression of *acs-2*, which encodes fatty acyl-CoA synthetase that acts in the initial stage of β-oxidation and catalyzes the conversion of fatty acids into fatty acyl-CoA esters. As presented in Figure 5A,B, the fluorescent signal of WBM170 administered with matrine was higher than that of untreated nematodes, suggesting that matrine upregulates the expression of *acs-2*. *acs-2* is known to be an important target gene of NHR-49 in regulating mitochondrial β-oxidation [26]. Thus, matrine inhibits high-glucose diet-induced fat accumulation by activating NHR-49 and inducing the expression of β-oxidation-associated genes, resulting in enhanced fatty acid β-oxidation in mitochondria.

Since the inhibition of delta-9 fatty acid desaturation enzymes such as FAT-7 is associated with reduced fat levels [25,26], we used transgenic DMS441 expressing *fat-7p::fat-7::GFP* reporter to analyze the effect of matrine on the transcription activity of *fat-7*. As presented in Figure 5C,D, the fluorescent signal in DMS441 administered with matrine was lower than that of untreated worms, implying that matrine can downregulate the expression of *fat-7*. To further confirm the role of *fat-7* in matrine′s fat lowering, we used the BX153 strain, a *fat-7* mutant, to analyze the effect of matrine on fat accumulation of high-glucose-diet BX153 by Oil red O staining. As expected, the fat-lowering effect of matrine was suppressed in *fat-7* mutant nematodes (Figure 5E,F), indicating that FAT-7 may be involved in the fat-lowering effect of matrine. Taken together, matrine could reduce fat accumulation by increasing fatty acid oxidation and inhibiting fatty acid synthesis in high-glucose-diet nematodes.

### 2.6. Matrine Impacts the Functions of Mitochondria and Peroxisomes

**Figure 6:**
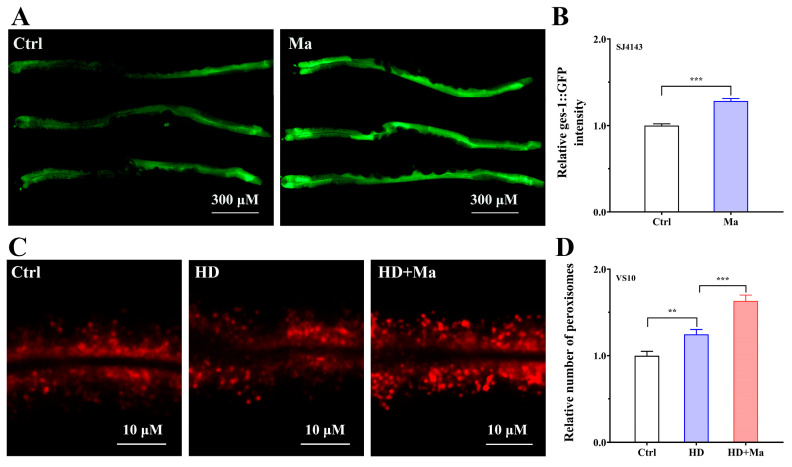
Matrine impacts the functions of mitochondria and peroxisomes. (**A**) Fluorescent images of SJ4143 (*ges-1::GFP(mit)*) nematodes. L1-stage SJ4143 were fed for 24 h and then placed on new plates with matrine (0 and 1.0 mM) for 48 h, and the fluorescent signal was observed. (**B**) Quantitative analysis of *ges-1::GFP* fluorescent intensity. (**C**) Fluorescent images of VS10 (*vha-6p::mRFP-PTS1*) nematodes. L1-stage VS10 nematodes were synchronized and fed for 24 h, then transferred to normal or high-glucose-diet NGM plates with matrine (0 and 1.0 mM) for 48 h to count the number of peroxisomes. (**D**) Fold change in the number of peroxisomes. Results are presented as mean ± SEM (*n* = 50, two independent trials). **: *p* < 0.01, ***: *p* < 0.001.

Studies reveal that AMPK is the critical regulator in modulating mitochondrial biogenesis and mitochondrial fission/fusion, in which NHR-49 may indirectly impact the structure and function of mitochondria by regulating fatty acid catabolism [27,28]. Since matrine reduced fat accumulation via the AAK-2/NHR-49 pathway, we used the transgenic SJ4143 nematodes that expressing the *ges-1::GFP(mit)* reporter to investigate the effect of matrine on mitochondria [29]. As presented in Figure 6A,B, matrine treatment enhanced the mitochondrial GFP intensity in intestinal cells, suggesting that activation of the AAK-2/NHR-49 pathway by matrine increases mitochondrial biogenesis and impacts mitochondrial function. In addition, peroxisomes are also closely related to lipid droplets and their capacities to carry out fatty acid decomposition and the detoxification of glyoxylate, particularly in efficiently decomposing long-chain and ultra-long-chain fatty acids [30]. Therefore, the transgenic VS10 nematodes (*vha-6p::mRFP-PTS1*) were used to investigate the effect of matrine on peroxisome biogenesis and function. The results showed that a high-glucose diet could significantly increase the number of peroxisomes (Figure 6C), possibly reflecting the changes in metabolic state after a high-glucose diet. However, it was worth noting that matrine treatment further increased the number of peroxisomes (Figure 6D). It is speculated that matrine promotes fatty acid β-oxidation by activating the AAK-2/NHR-49 pathway, thus causing an increase in the number of peroxisomes. Taken together, our study suggests that matrine activates the AAK-2/NHR-49 pathway, thereby affecting the biogenesis and function of mitochondria and peroxisomes.

### 2.7. Maintenance of AAK-2/NHR-49-Governed Lipid Homeostasis Increases the Physical Fitness and Lifespan

**Figure 7:**
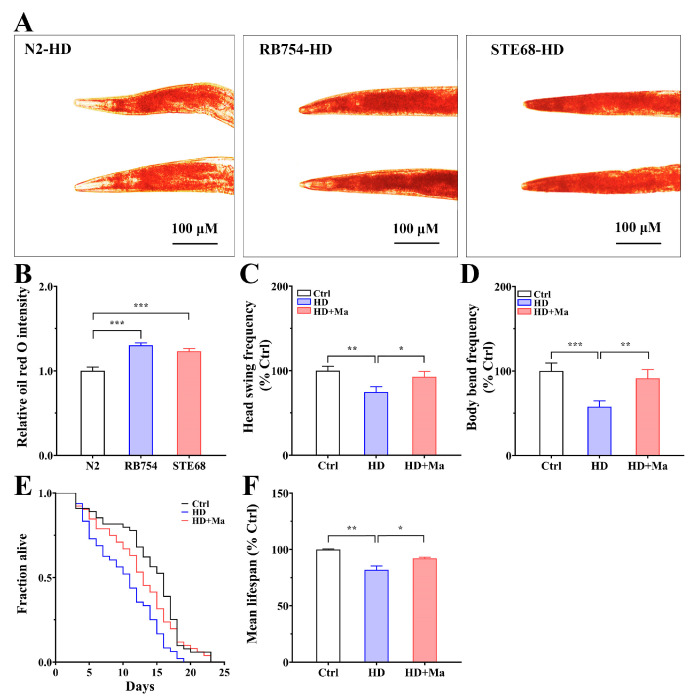
Maintenance of AAK-2/NHR-49-governed lipid homeostasis increases the physical fitness and lifespan in high-glucose-diet *C. elegans*. (**A**) Oil red O staining of N2, RB754 (*aak-2* mutant *(ok524*)), and STE68 (*nhr-49* mutant (*nr2041*)) nematodes. L1-stage N2, RB754, and STE68 nematodes were synchronized and fed on high-glucose-diet NGM plates for 72 h. (**B**) Quantification of fat content. Results are presented as mean ± SEM (*n* = 50 two independent trials). (**C**,**D**) Effects of matrine on head swings and body bends in N2 nematodes. L1 nematodes were fed for 24 h and then placed on normal or high-glucose-diet NGM plates with matrine (0 and 1.0 mM) for 48 h. Results are presented as mean ± SEM (*n* = 30 two independent trials). (**E**,**F**) Effects of matrine on lifespan in high-glucose diet. L1-stage N2 were fed for 24 h, and then placed on normal or high-glucose-diet NGM plates with matrine (0 and 1.0 mM). At 42 h, these nematodes were placed on new NGM plates with an additional 0.03 mg/mL FUdR to prevent spawning. The alive nematodes were calculated every 24 h (≈50 worms/group). *: *p* < 0.05, **: *p* < 0.01, ***: *p* < 0.001.

As presented in Figure 7A,B, a quantitative analysis of staining intensity shows that RB754 and STE68 mutants have higher fat content than N2 nematodes in a high-glucose diet, especially in *aak-2* mutants with excessive fat storage. The results suggest that the mutations of *aak-2* and *nhr-49* destroy lipid metabolic homeostasis, which may be accompanied by a decrease in the rate of fatty acid decomposition and an increase in the synthetic rate of fatty acids, leading to fat accumulation and obesity. In summary, the results suggest that AAK-2 and NHR-49 are essential regulators in maintenance of lipid homeostasis, which involves lipid storage and utilization. There are a number of health indicators to assess the physiological status of *C. elegans*, including locomotor-related parameters such as head swings and body bends [31]. Since matrine reduced fat content via the activation of the AAK-2/NHR-49-governed metabolic pathway, we further investigated whether it could improve obesity-related phenotypes in high-glucose-diet *C. elegans*. Through examining the locomotory parameters, we found that the locomotory indexes were decreased in high-glucose-diet nematodes, with the head swings reduced to 74.7% and body bends reduced to 57.6% (Figure 7C,D). In contrast, the high-glucose-diet nematodes treated with matrine significantly improved the locomotory indexes, with the head swings increased to 124.1% and body bends increased to 158.61%. These results indicate that matrine has the capacity for improving physical fitness and thus alleviating obesity-associated frailty in nematodes. According to the above experiments, matrine not only reduced fat accumulation, which is closely associated with obesity, but also increased health indicators, which reflects physical fitness status in high-glucose-diet *C. elegans*. Thus, we investigated whether matrine could promote longevity in a high-glucose diet. As presented in Figure 7E,F, the lifespan of N2 nematodes was prolonged by matrine, indicating the lifespan extension effect of matrine in high-glucose-diet *C. elegans*.

### 2.8. Matrine Alleviates Aβ-Mediated Lipid Metabolic Disorder

**Figure 8:**
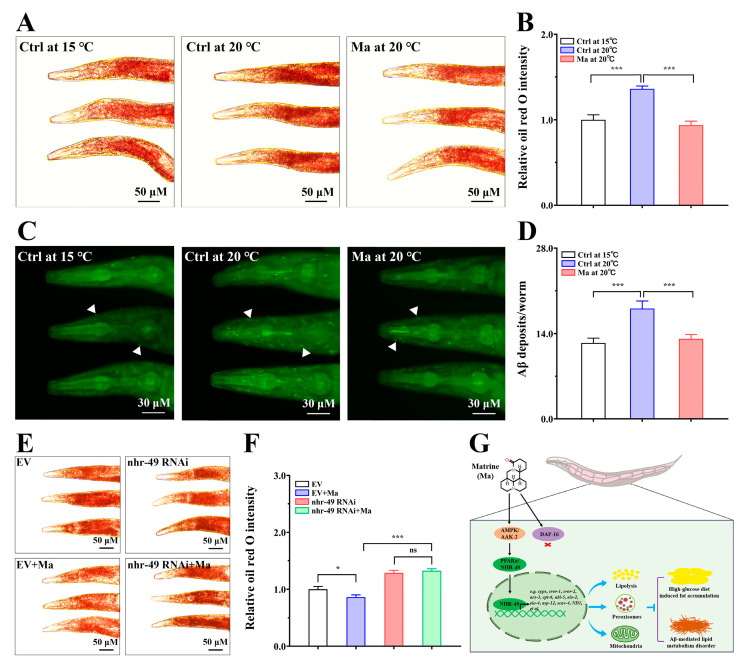
Matrine alleviates Aβ-mediated lipid metabolic disorder in *C. elegans* AD model. (**A**) Oil red O staining of CL2006 (*unc-54/Aβ1-42*). (**B**) Quantification of fat content. (**C**) ThS-stained images of CL2006. The white arrows indicate to Aβ deposits. (**D**) Quantification of Aβ deposits. L1-stage CL2006 nematodes were fed at 15 °C for 24 h and then placed on NGM plates with matrine (0 and 1.0 mM) at 20 °C for 48 h. Meanwhile, a group of nematodes cultured continuously at 15 °C served as control. Results are presented as mean ± SEM (*n* = 50 two independent trials). (**E**) Oil red O staining of CL2006 nematodes administered with or without *nhr-49* RNAi bacteria. L1-stage CL2006 nematodes were incubated on NGM plates containing matrine (0 and 0.1 mM), and fed with HT115 containing empty *L4440* or *nhr-49* recombinant at 20 °C for 72 h. (**F**) Quantification of fat content. Results are presented as mean ± SEM (*n* = 50, two independent trials). ns: not significant, *: *p* < 0.05, ***: *p* < 0.001. (**G**) Mechanism diagram of matrine in inhibiting high-glucose-diet-induced fat accumulation and Aβ-mediated lipid metabolic disorder via AAK-2/NHR-49 pathway in *C. elegans*.

AD is an age-associated neurodegenerative disorder accompanied by gradual memory loss and behavioral abnormalities, which is characterized by Aβ plaques and Tau neurofibrillary tangles [32]. In addition to these pathological hallmarks, abnormality in lipid metabolism has been considered another pathological characteristic of AD, which is related to pathogenesis and progression [33]. For instance, a study on the metabolite profiles of the cerebrospinal fluid of patients with AD found that lipid metabolic disorder was directly bound up with neuronal degeneration and memory decline [34]. Surprisingly, we found that Aβ aggregation toxicity induced lipid metabolic disorder in the AD nematode model CL2006, which is temperature-sensitive and expresses Aβ_1–42_ at 20 °C in its body wall muscle cells (Figure 8A,B). Since matrine could reduce fat accumulation and promote physical fitness and longevity in high-glucose-diet *C. elegans*, we investigated whether matrine could inhibit Aβ-induced lipid metabolic disorder in an AD nematode model. As expected, the fat accumulation in CL2006 nematodes was significantly reduced after treatment with matrine for 2 days as compared to that in the high-glucose-diet control, a 30.9% decrease (Figure 8A,B), demonstrating the alleviation effect on lipid metabolic disorder.

Aβ peptides undergo a series of morphological changes from their production to the formation of Aβ oligomers and Aβ fibers, and the Aβ oligomers have strong neurotoxicity, which is considered to be the main factor leading to cognitive dysfunction and neurodegeneration in AD [34,35]. Although the causal relationship between Aβ and AD has been controversial, Aβ aggregation and toxicity are undoubtedly some of the key factors driving the development of AD [36]. The Aβ-transgenic CL2006 generates Aβ plaques after the incubation temperature increases from 15 °C to 20 °C, which is often used to analyze Aβ deposition in the pharyngeal bulb [37]. Therefore, we tested whether matrine has the capacity to reduce Aβ deposits in the AD model. As presented in Figure 8C,D, a small amount of Aβ deposition in CL2006 nematodes could be observed when continuously incubated at 15 °C. When the temperature upshifted from 15 to 20 °C, the Aβ deposits per nematode were increased markedly. However, we found that matrine could decrease Aβ deposition, with a reduction in Aβ deposits to 72.6% (Figure 8C,D), implying the possible inhibitory effects of matrine on the aggregation of Aβ.

Numerous studies show that PPARα is not only a major metabolic regulator of energy homeostasis by controlling fatty acid oxidation in mitochondria and peroxisomes, but also has potentially neuroprotective effects, including the clearance of toxic Aβ peptides and the improvement of neurodegeneration and cognitive dysfunction [38]. It was reported that the activation of NHR-49/PPARα could reduce Aβ-mediated paralysis in a transgenic AD nematode model [39]. Therefore, we investigated whether matrine’s inhibition of Aβ-mediated lipid metabolic disorder is dependent on NHR-49. To investigate this, the transgenic AD model was used to conduct RNAi by feeding *E. coli* HT115 expressing double-stranded *nhr-49* RNA. As presented in Figure 8E,F, matrine could alleviate Aβ-mediated lipid metabolic disorder when using the HT115 containing empty *L4440* vector, but the mitigation of Aβ-mediated lipid metabolic disorder disappeared when feeding the recombinant *nhr-49* RNAi bacteria, indicating that the inhibition of Aβ-mediated lipid metabolic disorder might depend on NHR-49. Taken together, our study suggests that the activation of NHR-49/PPARα may be a promising strategy for the improvement of Aβ-induced lipid metabolic disorder and the inhibition of Aβ aggregation.

## 3. Discussion

Obesity occurs as a result of superfluous energy intake, leading to adipose deposition and metabolic disorder [1,3]. Current therapeutic strategies for obesity, such as dietary intervention and medications, are far from satisfactory, as they usually confront nutritional imbalance and the efficacy or safety of medications [3]. Thus, natural products with structural and functional diversities have garnered attention as promising alternatives in fighting against obesity [40]. Matrine, a naturally occurring quinoline alkaloid, is primarily sourced from leguminosae *Sophora flavescens*, which is usually selected to treat diabetes and obesity in traditional Chinese medicine. Feng et al. revealed that matrine upregulated LDL-receptor expression in in vitro cells, and further confirmed its role in regulating lipid metabolism by enhancing LDL uptake [16]. Though matrine has been reported to alleviate lipid metabolic disorder in hamsters [16], the mechanisms of matrine improving obesity and lipid metabolic disorder are rarely clarified in vivo. In this study, we constructed a high-glucose-diet-induced fat-accumulating *C. elegans* model, and found that matrine could reduce the fat content and the DHS-3::GFP-labeled lipid droplets in high-glucose diet N2 and transgenic LIU1 nematodes, respectively. To further investigate the mechanisms underlying matrine’s modulation of lipid metabolism, transcriptome analysis upon matrine treatment was performed in high-glucose-diet wild-type N2. The enrichment analysis of DEGs shows that the genes and pathways are related to glycolipid metabolism, detoxification, longevity, peroxisome, lysosome, and oxidative phosphorylation. Notably, a significant proportion of DEGs showed correlation with lipid metabolism, and most of these gene promoters contained NHR-49 binding sites. Similar to this result, Feng et al., employing quantitative proteomics in matrine-treated HepG2 cells, found that the upregulated proteins were enriched in cholesterol metabolism, lysosome, and thermogenesis [16]. These analyses suggest that matrine may stimulate lipolysis via regulating the activity of transcription factors and the expression of lipid-related target genes.

Abundant evidence shows that AMPK is the critical regulator that senses the energy state, and its activation contributes to improving lipid metabolism, reducing inflammation, and prolonging health lifespan [41,42]. It was reported that berberine, a natural alkaloid, increased the expression of adipotriglyceride lipase and stimulated basal lipolysis in adipocytes through a coupling mechanism associated with the AMPK pathway [8,9,43]. Our study found that loss of the AAK-2 function mutant suppressed the fat-lowering effect of matrine, and the hyperactivated AAK-2 strain had a relatively lower fat content than wild-type N2 in a high-glucose diet, suggesting that AAK-2 is involved in the matrine-mediated lipid-lowering effect. Emerging research shows that the responsiveness of AMPK gradually declines with aging. Nutrient overload also impairs AMPK activity and concurrently increases insulin resistance, thus promoting the appearance of lipid metabolic disorders like obesity [11]. Therefore, it is conceivable that matrine has the ability to activate AAK-2, which in turn impacts downstream effectors responsible for lipid homeostasis, and thereby ameliorates the dysfunction of lipid metabolism. It is well known that the IIS pathway senses nutrient or metabolite changes to regulate individual growth and metabolism [44]. However, our results showed that the inhibition of high-glucose-diet-induced fat accumulation was present in *daf-16* mutants, suggesting that the lipid-lowering effect of matrine is independent of DAF-16.

NHR-49, a homolog of PPARα in *C. elegans*, modulates gene expression profiles to synergically implement lipolysis or lipogenesis upon different nutritional states [24,45]. We observed that matrine increased the proportion of intestinal cells displaying nuclear localization of the NHR-49::GFP signal in PMD150 nematodes, and *nhr-49* RNAi not only eliminated the lipid-lowering effect of matrine, but also led to more significant fat accumulation, suggesting the involvement of NHR-49 in the matrine-mediated fat-lowering effect. Previous studies indicated that matrine reduced obesity through suppressing lipid synthesis and increasing fatty acid oxidation in high-fat-diet mice [15]. We speculate that matrine-activated NHR-49 induces the expression of target genes involved in decomposing fatty acids into acetyl-CoA, thereby producing energy in mitochondria [46]. Thus, transgenic WBM170 nematodes were used to analyze the expression of *acs-2*, which participates in the initial stage of fatty acid oxidation. Our study demonstrated that matrine could upregulate the expression level of *acs-2* in WBM170 nematodes. In addition, stearoyl-CoA desaturases, such as FAT-7, were found to exert an important role in the conversion of stearic acid into oleic acid, which is the substrate for the generation of triacylglycerol [47]. Our study found that matrine downregulated the expression of *fat-7* in transgenic DMS4441 nematodes, and the inhibition of matrine on fat accumulation was eliminated in the loss of function of the *fat-7* mutant, suggesting that inhibiting the production of oleic acid is one of the ways for matrine to reduce fat accumulation. In summary, these studies indicate that the regulation of lipid metabolism upon AMPK activation requires NHR-49, and subsequently impacts the expression of genes responsible for encoding enzymes linked to fat storage and breakdown in *C. elegans*.

Studies show that the AMPK signal influences mitochondrial homeostasis, such as mitochondrial biogenesis and mitochondrial fission/fusion, in response to metabolic changes [28]. Therefore, we selected the mitochondrial GFP-labeled strain, SJ4143 (*ges-1::GFP(mit)*), to measure GFP intensity as a product of mitochondrial accumulation [29]. The results showed that matrine treatment enhanced mitochondrial fluorescence, implying a corresponding increase in mitochondrial biogenesis [48]. In addition to mitochondria, fatty acid oxidation is also one of the basic functions of peroxisomes in living organisms [49]. Our study found that matrine supplementation not only upregulated the expression of *acox-1.2*, which is involved in fatty acid decomposition in peroxisomes, but also increased the number of peroxisomes in VS10 *C. elegans* (*vha-6p::mRFP-PTS1*), implying the promotion of peroxisome biogenesis and function. It was reported that the activation of AMPK prolonged lifespan through remolding mitochondrial homeostasis and promoting fatty acid oxidation in coordination with peroxisomes [50]. Therefore, we speculated that matrine might change the functions of mitochondria and peroxisome through the AAK-2/NHR-49-governed metabolic network, thus promoting fatty acid oxidation and reducing fat accumulation. Intriguingly, supplementing with natural products is recently reported to have benefits for physical fitness and lifespan through activating the AMPK pathway and mitochondrial dynamics [51,52]. In the present study, matrine was demonstrated to preserve physical fitness and extend lifespan in the fat-accumulating nematodes. In sum, we show the roles of matrine in the control of lipid metabolism in nematodes via the AAK-2/NHR-49 pathway, which is an important part of metabolic homeostasis, coordinating the system to adapt to energy challenges and ensuring the stability of lipid metabolism, thereby promoting health.

Lipid metabolic disorder is an important aspect of homeostasis imbalance in AD, which exacerbates the abnormal aggregation of Aβ and Tau and accelerates disease progression toward AD dementia [53]. Surprisingly, we found that Aβ toxicity also induced lipid metabolic disorder in a *C. elegans* AD model (Figure 8A,B). Since matrine regulates lipid metabolism through activation of the AAK-2/NHR-49 pathway, we investigated whether matrine could mitigate Aβ aggregation and toxicity in *C. elegans*. The study found that matrine not only alleviated lipid metabolic disorder caused by Aβ toxicity but also inhibited Aβ aggregation (Figure 8B,D). Recent studies demonstrated that natural products could initiate signaling cascades, which usually leads to the activation of transcription regulators and induces the expression of target genes to maintain homeostasis [54,55]. It was reported that matrine alleviated cognitive impairments in AD mice via the suppression of Aβ aggregation and the blockade of RAGE/Aβ axis [16]. In our works, studies using *nhr-49* RNAi showed that the inhibitory effect of matrine on Aβ-mediated lipid metabolic disorder may depend on the NHR-49 transcription factor. As in the above study, Leiteritz et al. reported that fenofibrate, a PPARα agonist used as a hypolipidemic drug, alleviated the paralytic phenotype induced by Aβ toxicity via activating the NHR-49/PPARα pathway in a *C. elegans* AD model [39]. Therefore, we infer that matrine inhibits Aβ-mediated lipid metabolic disorder through activation of the AAK-2/NHR-49 pathway, thereby altering the expression of lipid metabolic genes to re-establish homeostasis. Of course, Aβ-mediated lipid metabolic disorder is worthy of further study, especially to explore the mechanism of the relationship between Aβ aggregation toxicity and metabolic disorder. In summary, the present research not only offers new insights into the neuroprotective potential of matrine but also highlights its promise as a lipid-lowering agent with therapeutic potential for AD.

## 4. Materials and Methods

### 4.1. Reagents and C. elegans

Matrine (≥98%) and 5-fluoro-2′-deoxyuridine (FUdR) were bought from Aladdin (Shanghai, China). Cholesterol, ampicillin sodium, isopropyl β-D-thiogalactopyranoside (IPTG), and isopropanol were supplied by Macklin (Shanghai, China). The sodium azide (NaN_3_), Oil red O, and Thioflavin S (ThS) were provided by Sigma Chemical Corp. (St. Louis, MO, USA). Matrine stock solution (40 mM) was dissolved in S Medium, and then diluted appropriately for each experiment.

The *C. elegans* strains utilized in this work included N2, LIU1 (*dhs-3p::dhs-3::GFP*), RB754 (*aak-2(ok524)*), WBM60 (*aak-2p::aak-2(genomic aa1-321)::GFP*), TJ356 (*daf-16p::daf-16a/b::GFP*), CF1038 (*daf-16(mu86)*), PMD150 (*nhr-49p::nhr-49::GFP*), ABR161 (*vha-6p::mRFP-PTS1*; *dhs-3p::dhs-3::GFP*), WBM170 (*acs-2p::GFP*), DMS441 (*fat-7p::fat-7::GFP*), BX153 (*fat-7(wa36)*), SJ4143 (*ges-1::GFP(mit)*), VS10 (*vha-6p::mRFP-PTS1*), STE68 (*nhr-49(nr2041)*), and CL2006 (*pCL12(unc-54/human Aβ_1-42_)*) (Table S2). These worms were bought from Caenorhabditis Genetics Center (CGC) and incubated on nematode growth medium (NGM) plates with *Escherichia coli* OP50. To acquire age-synchronized L1-staged nematodes, the mature gravid nematodes were subjected to treatment with sodium hypochlorite, and the collected eggs were incubated in M9 buffer. When necessary, *C. elegans* were transferred to NGM plates containing 10 mM glucose and used as the high-glucose diet (HD) groups [19].

### 4.2. Body Length and Brood Size

In order to study the impact of matrine on *C. elegans* growth and brood size, body length and brood size assays were conducted on N2 nematodes [56]. In the body length assay, L4 nematodes were grown on NGM plates containing the indicated concentrations of matrine (0, 0.2, 0.5, 1.0, and 2.0 mM) and cultured for 48 h. The worms were randomly selected, and their images were acquired using an inverted microscope (Microshot Technology Co., Ltd., Guangzhou, China).

For the brood size assay, N2 nematodes were synchronized and grown on NGM solid plates until the L4 stage. Five nematodes per group were placed on the new NGM plates containing the indicated concentrations of matrine (0, 0.2, 0.5, 1.0 and 2.0 mM). They were transferred every day until the end of egg-laying. The plates with eggs were hatched for 24 h, and the quantity of progeny for each concentration was counted.

### 4.3. Fat Staining

The fat content of *C. elegans* was measured through Oil red O staining, and the protocol was slightly adjusted [18]. In brief, the *C. elegans* strains (including N2, RB754, WBM60, CF1038, BX153, STE68, and CL2006) were collected and washed three times with PBS containing 0.1% Triton X-100. These worms were dehydrated in 40% isopropanol for 3 min and stained in Oil red O solution prepared from 60% Oil red O stock solution for 2 h. Stained nematodes were placed on a slide and images were captured using an inverted microscope. Staining intensity was quantified using the ImageJ 1.53 software.

### 4.4. Fluorescent Signal Detection

To explore the effect of matrine on lipid metabolism, we selected transgenic strains (including LIU1, WBM170, and DMS441) to detect the fluorescent signal in matrine-treated nematodes. Briefly, L1-stage nematodes were fed for 24 h and subsequently put on NGM plates containing matrine (0 and 1.0 mM, respectively) for 48 h unless otherwise indicated. After being anesthetized with 1% NaN_3_, the GFP signal was observed with blue light (460~490 nm) by an inverted fluorescent microscope (MF52, Microshot Technology Co., Ltd., Guangzhou, China) and then the fluorescence values in individual worms were counted.

### 4.5. RNA Sequencing and Transcriptome Analysis

In order to clarify the transcription changes related to the lipid-lowering effects of matrine, RNA-seq was conducted on high-glucose-diet N2. Briefly, synchronized N2 nematodes were put on high-glucose-diet NGM plates with or without matrine (1.0 mM). After incubation for 48 h, worms were rapidly gathered and washed with M9 buffer 3 times, then flash-frozen in liquid nitrogen and kept at −80 °C until RNA extraction. Oligo (dT) magnetic beads were employed to enrich mRNA, which was then utilized to synthesize cDNA with random hexamer primers. After PCR amplification of the fragmented libraries, the quality-qualified libraries were subjected to high-throughput sequencing using Illumina (Metware, Wuhan, China). Differentially expressed genes (DEGs) were identified by selecting the false discovery rate (FDR) <  0.05 and fold change  (FC) ≥ 2 as the screening criteria. In order to analyze the targets of NHR-49, the JASPAR transcription factor database was used to detect the presence of consensus NHR-49 binding motif (Figure S1) at 2.0 kb upstream of DEGs [57]. The NHR-49 binding sites in the designated DEG promoter regions are shown in Supplementary Table S1.

### 4.6. NHR-49 Nuclear Localization Assay

The transgenic PMD150 strain expressing the *nhr-49p::nhr-49::GFP* reporter was used to evaluate the nuclear localization of NHR-49 [58]. In brief, L1 nematodes were grown for 24 h on NGM plates, then put on fresh NGM plates administered with or without matrine (1.0 mM) for 48 h. After being anesthetized with 1% NaN_3_, the PMD150 worms were observed and the GFP signal of NHR-49 in intestinal epithelia was counted. NHR-49::GFP was regarded as nuclear-localized when the fluorescence intensity of the GFP signal in the nucleus was at least twice that in the surrounding cytoplasm. The percentage of nuclear NHR-49::GFP was counted by dividing the number of intestinal cells in PMD150 nematodes.

### 4.7. DAF-16 Nuclear Localization Assay

To examine the impact of matrine on DAF-16, we selected TJ356 (*daf-16p::daf-16a/b::GFP*) nematodes to detect the nuclear translocation of this transcription factor. Briefly, L1-stage TJ356 nematodes were placed on NGM plates for 24 h. These worms were harvested and put on NGM plates supplemented with 0 or 1.0 mM matrine and further incubated for 48 h. After being anesthetized with 1% NaN_3_, the GFP signal of DAF-16 was observed using a Mshot MF52 fluorescence microscope (Microshot Technology Co., Ltd., Guangzhou, China). Approximately 25 nematodes per trial were randomly chosen to assess subcellular localization of DAF-16::GFP, and the fraction of nematodes in the “cytoplasm”, “intermediate”, and “nucleus” was counted. This experiment was performed two times to obtain similar results. 

### 4.8. RNA Interference

To clarify the role of NHR-49 in matrine’s lipid lowering, nhr-49 RNAi was performed by feeding *E. coli* HT115 with some modifications [37]. For constructing the RNAi plasmid, a 904 bp *nhr-49* sequence was amplified, sequenced, and inserted into the *L4440* vector (primer-F: TGCTCTAGATACCGAAGATCGCAAATC and primer-R: CGGGGTACCCCATTAGTCGGTAACGT). L1-stage N2 and ABR161 nematodes were fed with HT115 carrying empty *L4440* or the *nhr-49* recombinant for 24 h, then put on high-glucose plates coated with HT115 (containing *L4440* or *nhr-49* recombinant plasmid) and administered with matrine (0 and 1.0 mM) for 48 h. For CL2006, L1-stage nematodes were fed with HT115 carrying empty *L4440* or the *nhr-49* recombinant, and administered with matrine (0 and 1.0 mM) for 72 h. These nematodes were collected for staining and photographing to analyze the role of NHR-49 in the lipid-lowering effect of matrine.

### 4.9. Quantification of Mitochondria and Peroxisomes

As previously described [29], we used the transgenic SJ4143 (*ges-1::GFP(mit)*) to measure GFP intensity as a product of mitochondrial accumulation. In brief, L1-stage SJ4143 nematodes were cultured on NGM plates for 24 h and then put on new NGM plates containing matrine (0 and 1.0 mM, respectively) for 48 h. After being anesthetized with 1% NaN_3_, the mitochondrial GFP intensity of SJ4143 nematodes was photographed by a Mshot MF52 fluorescence microscope. Moreover, to explore the effect of matrine on peroxisomes, we employed VS10 worms (*vha-6p::mRFP-PTS1*) to observe the changes in peroxisomes in matrine-treated nematodes [59]. L1-stage VS10 nematodes were placed on NGM plates for 24 h, and transferred to normal or high-glucose-diet NGM plates with or without matrine (1.0 mM) for 48 h. The anesthetized nematodes were photographed and used for quantifying the number of peroxisomes. These experiments were performed two times to obtain similar results.

### 4.10. Motility Analysis

To study the influence of matrine on the locomotion of high-glucose-diet *C. elegans*, we conducted locomotory experiments using wild-type N2. Briefly, L1-stage worms were cultivated for 24 h and then put on normal or high-glucose-diet NGM solid plates with or without matrine (0 and 1.0 mM, respectively) for 48 h. The nematodes were transferred to NGM plates lacking *E. coli* OP50 and settled for 1 min, then the frequencies of head swings and body bends within 30 s were separately recorded. Each head swing of the nematodes to the left and right was counted as one swing, and one body bend was defined as a sine wave formed along the body axis of the nematode in its moving direction.

### 4.11. Lifespan Assay

To study the impact of matrine on the lifespan of *C. elegans* with a high-glucose diet, we carried out lifespan experiments using wild-type N2. Briefly, L1-stage N2 nematodes were fed on NGM plates for 24 h, then transferred to normal or high-glucose-diet NGM agar plates (*N* ≈ 50 worms per group) with or without matrine (1.0 mM) for 18 h. Subsequently, the nematodes were shifted to normal or high-glucose-diet NGM plates containing 0.03 mg/mL FUdR and matrine (0 and 1.0 mM, respectively). The alive nematodes were counted every day, and the nematodes were transferred to new NGM agar plates every 24 h until all nematodes died.

### 4.12. ThS Staining Assay

The Aβ deposits in the *C. elegans* CL2006 strain were observed by ThS staining, and this method was slightly different from the previous description [37]. L1-stage CL2006 nematodes were incubated for 24 h at 15 °C, then put on new NGM plates supplemented with 0 or 1.0 mM matrine and cultivated at 20 °C for another 48 h. Meanwhile, a group of CL2006 nematodes cultured continuously at 15 °C was used as the control. After treatment, nematodes were collected using PBS and frozen at −80 °C for 15 min. The nematodes were stained with ThS solution (0.125% ThS and 50% ethanol) and soaked for 20 min. Subsequently, these worms were decolorized using 50% ethanol until the staining agent disappeared completely, and the anterior pharyngeal bulb images were captured with an MF52 inverted microscope.

### 4.13. Data Analysis

Analysis was performed using GraphPad Prism 8.0. A one-way analysis of variance (ANOVA) was employed to compare more than two groups. Pair-wise comparison was carried out using the *t*-test. Survival curve was performed using a Kaplan–Meier plot and was examined with the log-rank test. The DEGs were identified with the following criteria: FDR < 0.05 and FC ≥ 2.0. The JASPAR database was used to identify the NHR-49 binding sites in 2.0 kb upstream regions of DEGs, and the relative profile score threshold was set as 90%. The statistical *p* < 0.05 was defined as significant.

## 5. Conclusions

Our study shows that matrine reduces fat content and DHS-3::GFP-labeled lipid droplets in high-glucose-diet N2 and transgenic LIU1 nematodes, respectively. RNA-seq results show that the enriched signaling pathways are mainly involved in glycolipid metabolism, detoxification, longevity, peroxisome, lysosome, and oxidative phosphorylation. Further study found that the inhibition of fat accumulation by matrine was attributed to the activation of the AAK-2/NHR-49 signal pathway, thereby exerting lipid-lowering effects through enhancing fatty acid β-oxidation and inhibiting lipid synthesis. Using the transgenic SJ4143 (*ges-1::GFP(mit)*) and VS10 (*vha-6p::mRFP-PTS1*), the study showed that matrine activated the AAK-2/NHR-49 pathway coupling the alteration of mitochondrial and peroxisomal function. Moreover, studies of *aak-2* and *nhr-49* mutants suggest that AAK-2 and NHR-49 are the key regulators to modulate lipid homeostasis in *C. elegans,* and the *aak-2* mutant in particular leads to significantly increased fat storage. However, matrine supplements could increase the physical fitness and lifespan of high-glucose-diet nematodes. Additionally, we found that Aβ induced lipid metabolic disorders in a *C. elegans* AD model, and matrine not only reduced Aβ aggregation but also alleviated Aβ-mediated lipid metabolic disorder. Collectively, our findings indicate that matrine alters fat breakdown and storage via the AAK-2/NHR-49-governed metabolic pathway, which highlights its promise as a lipid-lowering agent but also offers preliminary insights into its neuroprotective potential with therapeutic implications for AD.

## Data Availability

The data presented in this study are available on request from the corresponding author.

## Supplementary Materials

The following supporting information can be downloaded at: https://www.mdpi.com/article/10.3390/ijms26073048/s1.
