# Supplementary material for: Matrine Inhibits High-Glucose-Diet-Induced Fat Accumulation and Aβ-Mediated Lipid Metabolic Disorder via AAK-2/NHR-49 Pathway in Caenorhabditis elegans
Source: Int J Mol Sci. 2025 Mar 26;26(7):3048. doi: 10.3390/ijms26073048 (PMC11988642; doi:10.3390/ijms26073048)
Supplement: Supplementary file 1 [file ijms-26-03048-s001.zip › Supplementary materials.pdf]

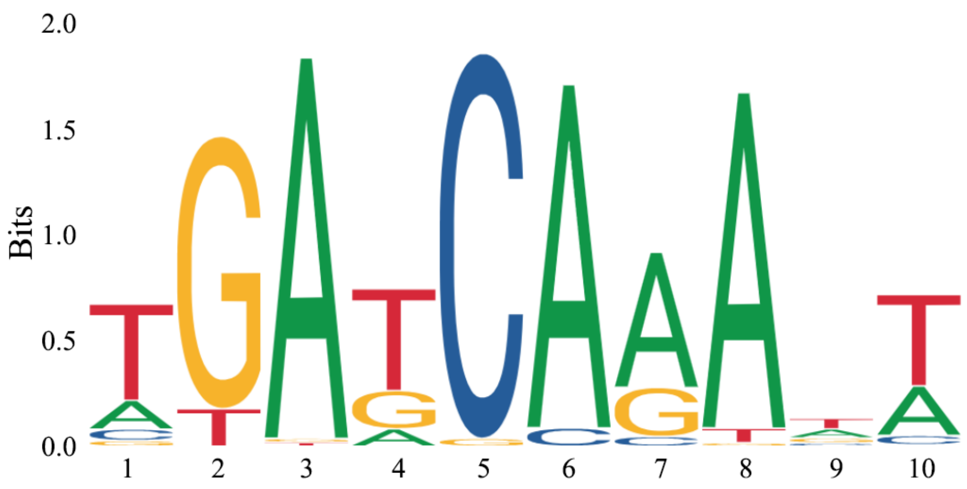

Figure S1 Consensus NHR-49-binding motifs. JASPAR are used to identify binding motifs and generate graphs, respectively. See also Table S1.

Table S1 JASPAR website predicted binding sites of NHR-49 to the promoter regions of the differential genes in the chordograms.

Gene name	Number of binding sites	Matrix ID	Name	Score	Relative score	Sequence ID	Start	End	Strand	Predicted sequence
cyp-35B3	2	UN0746.1	UN0746.1.nhr-49	12.243627	0.94156975	NC_003283.11:3943353-3945353	1410	1419	+	TTATCAAATT
		UN0746.1	UN0746.1.nhr-49	11.668645	0.9287818	NC_003283.11:3943353-3945353	912	921	+	AGATCAGAAT
cror-1	1	UN0746.1	UN0746.1.nhr-49	14.675479	0.9956555	NC_003280.10:c14589507-14587507	1835	1844	+	TGATCAAAT
cyp-35D1	3	UN0746.1	UN0746.1.nhr-49	11.539405	0.92590743	NC_003283.11:16067238-16069238	671	680	-	TGAACAAAAA
		UN0746.1	UN0746.1.nhr-49	10.795113	0.90935403	NC_003283.11:16067238-16069238	1724	1733	+	TGATCAATAT
		UN0746.1	UN0746.1.nhr-49	10.420488	0.90102214	NC_003283.11:16067238-16069238	658	667	+	ATATCAAAAT
cyp-33C6	1	UN0746.1	UN0746.1.nhr-49	10.885498	0.91136426	NC_003283.11:c2251410-2249410	1295	1304	+	AGAGCAAAAA
cyp-35A5	2	UN0746.1	UN0746.1.nhr-49	13.841189	0.9771004	NC_003283.11:3934326-3936326	719	728	+	TGATCAAAAA
		UN0746.1	UN0746.1.nhr-49	11.81804	0.93210447	NC_003283.11:3934326-3936326	715	724	-	TGATCAAAAC
cror-2	2	UN0746.1	UN0746.1.nhr-49	12.163887	0.9397963	NC_003283.11:c2769565-2767565	322	331	+	TGAGCAGATT
		UN0746.1	UN0746.1.nhr-49	11.539405	0.92590743	NC_003283.11:c2769565-2767565	1149	1158	-	TGAACAAAAA
ubl-5	3	UN0746.1	UN0746.1.nhr-49	13.29644	0.96498495	NC_003279.8:c5610841-5608841	1282	1291	-	TGATCAGAAT
		UN0746.1	UN0746.1.nhr-49	12.5690365	0.948807	NC_003279.8:c5610841-5608841	925	934	+	TGAACAAATT
		UN0746.1	UN0746.1.nhr-49	10.715757	0.9075891	NC_003279.8:c5610841-5608841	130	139	+	TGAACAAAGA

F08H9.4	3	UN0746.1	UN0746.1.nhr-49	12.048284	0.9372252	NC_003283.11:c14466428-14464428	325	334	+	TTATCAAAAT
		UN0746.1	UN0746.1.nhr-49	11.719788	0.9299193	NC_003283.11:c14466428-14464428	686	695	-	AGAGCAAAAT
		UN0746.1	UN0746.1.nhr-49	10.619056	0.90543836	NC_003283.11:c14466428-14464428	1691	1700	-	CGAGCAAAAT
acs-3	1	UN0746.1	UN0746.1.nhr-49	12.213394	0.9408974	NC_003283.11:1967528-1969528	1674	1683	-	AGATCAAAAA
asp-12	2	UN0746.1	UN0746.1.nhr-49	11.91513	0.93426377	NC_003283.11:8268643-8270643	896	905	-	AGAGCAAATT
		UN0746.1	UN0746.1.nhr-49	10.885498	0.91136426	NC_003283.11:8268643-8270643	381	390	+	AGAGCAAAAA
hpo-18	1	UN0746.1	UN0746.1.nhr-49	12.243627	0.94156975	NC_003283.11:c4348425-4346425	213	222	-	TTATCAAATT
aman-1	1	UN0746.1	UN0746.1.nhr-49	11.029698	0.9145713	NC_003284.9:4731095-4733095	1165	1174	+	AGATCAGATA
hsp-16.11	2	UN0746.1	UN0746.1.nhr-49	11.550047	0.9261441	NC_003283.11:9088077-9090077	13	22	-	TGAACAAAGT
		UN0746.1	UN0746.1.nhr-49	11.550047	0.9261441	NC_003283.11:9088077-9090077	1723	1732	+	TGAACAAAGT
cyp-33C5	1	UN0746.1	UN0746.1.nhr-49	11.224637	0.91890687	NC_003283.11:2243004-2245004	1484	1493	+	TTATCAAAGT
elo-4	2	UN0746.1	UN0746.1.nhr-49	12.657494	0.95077443	NC_003281.10:c9336274-9334274	1725	1734	-	TGATCAGATA
		UN0746.1	UN0746.1.nhr-49	10.864588	0.91089916	NC_003281.10:c9336274-9334274	1766	1775	+	TTATCAGATT
scav-4	6	UN0746.1	UN0746.1.nhr-49	12.373694	0.9444625	NC_003284.9:12979775-12981775	1124	1133	-	TGAACAAAAT
		UN0746.1	UN0746.1.nhr-49	12.224036	0.941134	NC_003284.9:12979775-12981775	55	64	-	AGATCAAAGT
		UN0746.1	UN0746.1.nhr-49	12.213394	0.9408974	NC_003284.9:12979775-12981775	998	1007	+	AGATCAAAAA
		UN0746.1	UN0746.1.nhr-49	11.638504	0.9281115	NC_003284.9:12979775-12981775	745	754	+	TGATCAGAGA

		UN0746.1	UN0746.1.nhr-49	11.389747	0.92257893	NC_003284.9:12979775-12981775	1729	1738	+	AGATCAAAGA
		UN0746.1	UN0746.1.nhr-49	10.634344	0.9057784	NC_003284.9:12979775-12981775	1551	1560	-	TGATCAGATC
		UN0746.1	UN0746.1.nhr-49	11.81804	0.93210447	NC_003282.8:c17469408-17467408	204	213	-	TGATCAAAAC
elo-2	3	UN0746.1	UN0746.1.nhr-49	11.81804	0.93210447	NC_003282.8:c17469408-17467408	580	589	-	TGATCAAAAC
		UN0746.1	UN0746.1.nhr-49	10.990456	0.91369855	NC_003282.8:c17469408-17467408	208	217	+	TGATCAATTT
		UN0746.1	UN0746.1.nhr-49	13.047684	0.95945245	NC_003283.11:c6495921-6493921	1754	1763	-	AGATCAAAAT
cpt-6	4	UN0746.1	UN0746.1.nhr-49	11.213994	0.9186701	NC_003283.11:c6495921-6493921	846	855	+	TTATCAAAAA
		UN0746.1	UN0746.1.nhr-49	11.144897	0.9171334	NC_003283.11:c6495921-6493921	1581	1590	-	TGAGCAGAGT
		UN0746.1	UN0746.1.nhr-49	10.567913	0.90430087	NC_003283.11:c6495921-6493921	1741	1750	+	CGATCAGAAT
ND1	1	UN0746.1	UN0746.1.nhr-49	14.870821	1	NC_001328.1:1-1763	922	931	-	TGATCAAATT
fat-7	1	UN0746.1	UN0746.1.nhr-49	10.634344	0.9057784	NC_003283.11:c7155146-7153146	1845	1854	+	TGATCAGATC
ND5	1	UN0746.1	UN0746.1.nhr-49	10.729308	0.90789044	NC_001328.1:9691-11691	1085	1094	+	TGATCAAATG
daf-2	2	UN0746.1	UN0746.1.nhr-49	13.841189	0.9771004	NC_003281.10:c3042846-3040846	1338	1347	+	TGATCAAAAA
		UN0746.1	UN0746.1.nhr-49	12.462151	0.94642985	NC_003281.10:c3042846-3040846	1334	1343	-	TGATCAGAAA
		UN0746.1	UN0746.1.nhr-49	13.841189	0.9771004	NC_003279.8:c8988540-8986540	940	949	-	TGATCAAAAA
fasn-1	3	UN0746.1	UN0746.1.nhr-49	10.745899	0.9082594	NC_003279.8:c8988540-8986540	196	205	-	AGAACAAAAT
		UN0746.1	UN0746.1.nhr-49	10.720387	0.9076921	NC_003279.8:c8988540-8986540	855	864	+	TTAGCAAAAT

dhs-25	4	UN0746.1	UN0746.1.nhr-49	13.347583	0.9661223	NC_003284.9:1484532-1486532	607	616	+	TGAGCAAAAT
		UN0746.1	UN0746.1.nhr-49	12.243627	0.94156975	NC_003284.9:1484532-1486532	1379	1388	+	TTATCAAATT
		UN0746.1	UN0746.1.nhr-49	11.213994	0.9186701	NC_003284.9:1484532-1486532	1717	1726	-	TTATCAAAAA
		UN0746.1	UN0746.1.nhr-49	10.795113	0.90935403	NC_003284.9:1484532-1486532	1973	1982	-	TGATCAATAT
cyp-14A5	4	UN0746.1	UN0746.1.nhr-49	12.243627	0.94156975	NC_003283.11:c5425282-5423282	410	419	+	TTATCAAATT
		UN0746.1	UN0746.1.nhr-49	12.048284	0.9372252	NC_003283.11:c5425282-5423282	1392	1401	+	TTATCAAAAT
		UN0746.1	UN0746.1.nhr-49	11.409337	0.9230147	NC_003283.11:c5425282-5423282	359	368	-	TTATCAAATA
		UN0746.1	UN0746.1.nhr-49	10.961909	0.9130636	NC_003283.11:c5425282-5423282	61	70	-	AGATCAAACA
nhr-79	8	UN0746.1	UN0746.1.nhr-49	13.841189	0.9771004	NC_003283.11:c19213895-19211895	589	598	+	TGATCAAAAA
		UN0746.1	UN0746.1.nhr-49	13.841189	0.9771004	NC_003283.11:c19213895-19211895	1752	1761	-	TGATCAAAAA
		UN0746.1	UN0746.1.nhr-49	13.542925	0.97046685	NC_003283.11:c19213895-19211895	1626	1635	-	TGAGCAAATT
		UN0746.1	UN0746.1.nhr-49	13.491783	0.9693295	NC_003283.11:c19213895-19211895	585	594	-	TGATCAGATT
		UN0746.1	UN0746.1.nhr-49	12.523935	0.947804	NC_003283.11:c19213895-19211895	1154	1163	-	TGAGCAAAGT
		UN0746.1	UN0746.1.nhr-49	11.946952	0.9349715	NC_003283.11:c19213895-19211895	1328	1337	+	CGATCAAAAT
		UN0746.1	UN0746.1.nhr-49	11.550047	0.9261441	NC_003283.11:c19213895-19211895	436	445	+	TGAACAAAGT
		UN0746.1	UN0746.1.nhr-49	11.224637	0.91890687	NC_003283.11:c19213895-19211895	495	504	+	TTATCAAAGT
rbc-2	1	UN0746.1	UN0746.1.nhr-49	10.399121	0.9005469	NC_003281.10:c2530689-2528689	1682	1691	+	TGATGAAATT

F42G2.7	1	UN0746.1	UN0746.1.nhr-49	12.5690365	0.948807	NC_003280.10:2416158-2418158	1058	1067	-	TGAACAAATT

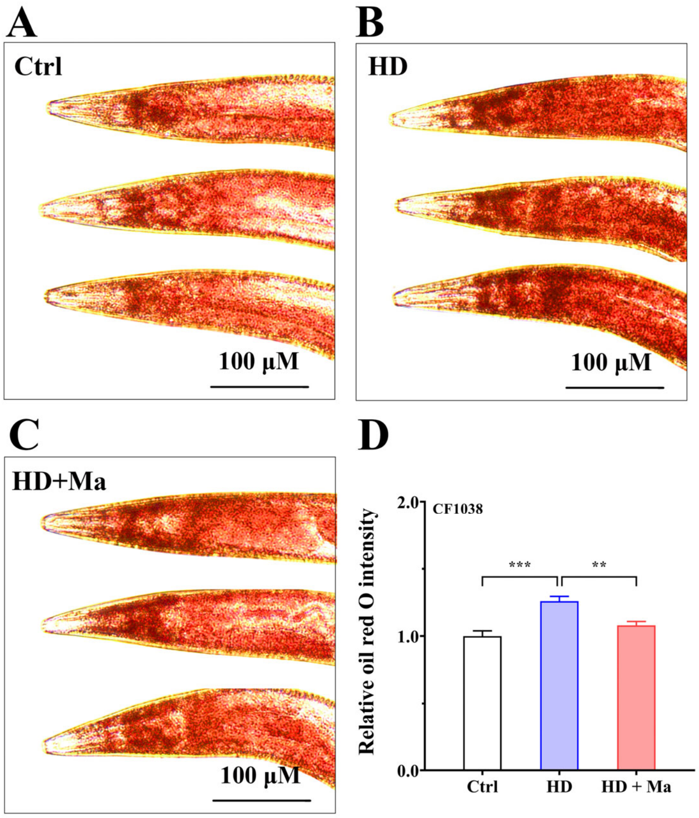

Figure S2 (A-C) Oil Red O staining of CF1038 (*daf-16* mutant (*mu86*)) nematodes. The CF1038 nematodes were synchronized and fed for 24 h, then transferred to normal or high-glucose diet NGM plates with or without matrine for 48 h. (H) Quantitative analysis of Oil Red O staining. Results are presented as mean ± SEM (n=50 two independent trials). **: $p < 0.01$, ***:$p < 0.001$.

Table S2 *C. elegans* strains

Strain	Genotype
ABR161	*vha-6p::mRFP-PTS1 + Cbr-unc-119(+)*
BX153	*fat-7(wa36) V*
CF1038	*daf-16(mu86) I*
CL2006	*pCL12(unc-54/human Abeta peptide 1-42 minigene) + rol-6(su1006)*
DMS441	*fat-7p::fat-7::GFP + lin15(+)*
LIU1	*dhs-3p::dhs-3::GFP + unc-76(+)*
N2	*wild type*
PMD150	*nhr-49p::nhr-49::GFP + myo-2p::mCherry*
RB754	*aak-2(ok524) X*
SJ4143	*ges-1::GFP(mit)*
STE68	*nhr-49(nr2041) I*
TJ356	*daf-16p::daf-16a/b::GFP + rol-6(su1006)*
VS10	*vha-6p::mRFP-PTS1 + Cbr-unc-119(+)*
WBM170	*acs-2p::GFP + rol-6(su1006)*
WBM60	*aak-2p::aak-2(genomic aa1-321)::GFP::unc-54 3'UTR + myo-2p::tdTOMATO*
